# Why does society accept a higher risk for alcohol than for other voluntary or involuntary risks?

**DOI:** 10.1186/s12916-014-0189-z

**Published:** 2014-10-21

**Authors:** Jürgen Rehm, Dirk W Lachenmeier, Robin Room

**Affiliations:** Centre for Addiction and Mental Health, 33 Russell Street, Toronto, ON M5S 2S1 Canada; Addiction Policy, Dalla Lana School of Public Health, University of Toronto, Toronto, Canada; Department of Psychiatry, Faculty of Medicine, University of Toronto, Toronto, Canada; Institute of Medical Science, University of Toronto, Toronto, Canada; Clinical Psychology and Psychotherapy, Technische Universität Dresden, Dresden, Germany; Chemisches und Veterinäruntersuchungsamt Karlsruhe, Karlsruhe, Germany; Centre for Alcohol Policy Research, Turning Point, Fitzroy, VIC Australia; Melbourne School of Population & Global Health, University of Melbourne, Melbourne, Australia; Centre for Social Research on Alcohol & Drugs, Stockholm University, Stockholm, Sweden

**Keywords:** Acceptable risk, Alcohol, Mortality, Patterns of drinking, Risk, Voluntary versus involuntary risk

## Abstract

**Background:**

Societies tend to accept much higher risks for voluntary behaviours, those based on individual decisions (for example, to smoke, to consume alcohol, or to ski), than for involuntary exposure such as exposure to risks in soil, drinking water or air. In high-income societies, an acceptable risk to those voluntarily engaging in a risky behaviour seems to be about one death in 1,000 on a lifetime basis. However, drinking more than 20 g pure alcohol per day over an adult lifetime exceeds a threshold of one in 100 deaths, based on a calculation from World Health Organization data of the odds in six European countries of dying from alcohol-attributable causes at different levels of drinking.

**Discussion:**

The voluntary mortality risk of alcohol consumption exceeds the risks of other lifestyle risk factors. In addition, evidence shows that the involuntary risks resulting from customary alcohol consumption far exceed the acceptable threshold for other involuntary risks (such as those established by the World Health Organization or national environmental agencies), and would be judged as not acceptable. Alcohol’s exceptional status reflects vagaries of history, which have so far resulted in alcohol being exempted from key food legislation (no labelling of ingredients and nutritional information) and from international conventions governing all other psychoactive substances (both legal and illegal). This is along with special treatment of alcohol in the public health field, in part reflecting overestimation of its beneficial effect on ischaemic disease when consumed in moderation.

**Summary:**

A much higher mortality risk from alcohol than from other risk factors is currently accepted by high income countries.

**Electronic supplementary material:**

The online version of this article (doi:10.1186/s12916-014-0189-z) contains supplementary material, which is available to authorized users.

## Background

Dealing with risk is a critical, complex and not always fully consistent endeavour in modern high-income societies [1,2]. This contribution will examine the way the risks associated with alcohol are handled, restricting our examinations to mortality and health risks. We first introduce the classic separation between involuntary and voluntary risks [3]. Voluntary risk is associated with activities in which individuals participate by choice, and where they use their own value system and experience to determine if the risk of a voluntary activity is acceptable to them. Examples are to smoke, to consume alcohol or to ski. Involuntary risks are associated with activities, conditions or events to which individuals might be exposed without their consent. Examples of involuntary risks include the risks of natural disasters (earthquakes, floods, and so on), or technology-related risks such as bad air quality or contaminated water. As Starr showed in his seminal paper [3], societies tend to accept much higher risks for voluntary behaviours than for involuntary exposure. The latter risks are often dealt with by special agencies such as the Environmental Protection Agency in the US or the European Environment Agency in Europe.

Voluntary risks are dealt with by a variety of means. These include total or partial prohibitions on commerce in risky behaviours, such as no tobacco sold to minors, as stipulated by the Tobacco Framework Convention [4]; heroin production and sale prohibited except for medical and scientific purposes [5] (both of which also remind us that some of the answers to minimizing risk go beyond national governments); or a minimal legal purchasing age for alcohol [6]. Or governments may gently discourage the behaviour with controls on availability or on price, for instance with Pigouvian taxes [7] (taxes applied to a market activity or product that is generating cost for individuals or society - so-called negative externalities), including taxes to channel consumption and behaviour for public health purposes [6]. But in many cases these risks are left to individual choice, and only education or guidelines are provided. However, exceptions will be made if lives of others are concerned. Thus, where behaviour such as drinking alcohol or smoking cigarettes results in involuntary risks to others, limits are often imposed, for example, interdiction of smoking in restaurants and public places in reaction to risks from involuntary smoking; *per se* laws to disallow operating machinery or driving after drinking. Certain risks are between voluntary and involuntary, such as the risk of participating in motor traffic (while some participation is voluntary, other participation is necessary for earning money; and some of the risk is from others’ behaviour), or risks associated with various food groups (for example, exposure to salmonella will be involuntary, but exposure to saturated fats listed on the label is considered voluntary). Such risks are usually regulated by specialized agencies, stipulating rules to limit risks by thresholds for ingredients in food (for example, the Joint Food and Agriculture Organization/World Health Organization (WHO) Expert Committee on Food Additives at the international level [8], the Food and Drug Administration (FDA) in the US, and the European Food Safety Authority in Europe), or by specific rules such as speed limits, safety belts or safety requirements for baby walkers or bunk beds - for instance, by the US Consumer Product Safety Commission.

**Figure 1:**
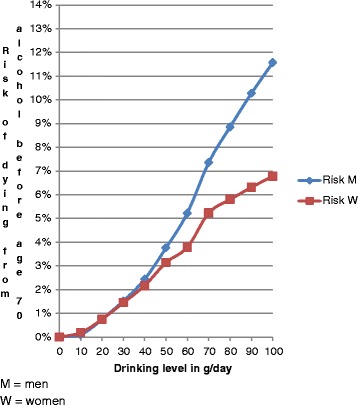
**Risk of dying prematurely (up to age 70) because of alcohol consumption.** Drinking level measured in grams of pure alcohol per day (average for six EU countries based on mortality profile for 2012).

Alcohol consumption is a major risk factor for mortality and burden of disease globally in most middle and high-income countries [6,9]. Figure 1 shows the average odds of dying from different levels of alcohol exposure up to age 70 for six countries of the European Union (EU). The countries were selected to include at least one from each of the three prototypical drinking pattern traditions in Europe [10-12]. Italy was selected to represent the wine‐drinking countries in the Mediterranean region, where wine is often consumed daily, usually with meals and avoiding drunkenness. Italy is also the second lowest consuming country in the EU [6], and has one of the lowest rates of alcohol-use disorders [13]. Ireland was selected as typical for the Central‐West and Western regions, with beer as the beverage of choice, and with higher consumption and proportionally less drinking with meals than in Italy. The level of consumption is close to the European average [6,14]. Four different countries were selected for Nordic and Central‐East and Eastern regions, which share a traditional style of irregular heavy drinking, mostly outside meals. The countries selected represent some variation in wealth and life expectancy [15,16]: for the Nordic countries, Finland was selected; Estonia was chosen as representative of the Central East/Eastern European EU countries with the lowest gross domestic product at purchasing power parity per capita and lowest life expectancy, with one of the highest rates of alcohol-use disorders in the EU [13]; Hungary as a medium wealthy country for this region with high alcohol consumption; and Poland as a relatively rich country with one of the longest life expectancies in this region.

The odds of dying from different levels of alcohol are stated in absolute-risk terms but are based on the level-specific relative risks applied to the mortality patterns of these countries in 2012, where the effects of current drinking had been subtracted and where competing risks had been removed (see Additional file 1 for details; for an overview of relative risks of alcohol by level of drinking, see [17,18]; for current burden of alcohol by country, see [6]; for competing risks, see [19]). The lifetime risk of dying was estimated assuming steady average daily alcohol consumption from age 15 and up. The difference in odds by gender is the net effect of the overall higher mortality risks of men compared to women at any age and for almost any causes, but particularly for injuries, moderated by the higher relative mortality risks for women than for men from biological actions of alcohol at any given level of drinking [17,18].

How do these risks compare to other acceptable risks in society? Many of the fully involuntary risks, such as unsafe water provided to a household, have risk thresholds set at one in one million (1 in 10^6^). Indeed, the one in one million has become something of a gold standard of acceptable risk for involuntary exposure and has been used in different areas such as water safety in Australia and the US [20,21], or for increases of exposure to carcinogens in air, sediment or soil [22]. It should be noted that other standards have been used, and sometimes we see ranges, such as one in a million to one in 100,000 (see also [23]). Starr [3] found that the public seems to be willing to accept voluntary risks roughly 1,000 times greater than involuntary risks. By this standard, an acceptable risk for voluntary risks experienced by the drinkers themselves is one in 1,000 deaths for the pattern of behaviour over a lifetime.

## Discussion

If we accept the stated acceptable risk of one in 1,000 deaths, drinking 20 g pure alcohol per day (equivalent to 1.5 to 2.5 standard drinks dependent on the national standard drink: 8 g pure alcohol per drink in the UK, between 10 and 14 g in other European countries) exceeds this threshold, even if only the risk up to age 70 is considered (obviously, the lifetime risks for alcohol-attributable mortality will be considerably higher). Although cause-of-death statistics become problematic for older ages [24], and thus the estimated risks are more uncertain, drinking 20 g pure alcohol per day seems to exceed a threshold of one in 100 for death on a lifetime basis. To put this in perspective, the average level of daily consumption in EU countries in 2012 was about 31 g pure alcohol per day among drinkers, entailing a mortality risk beyond this threshold. This level of drinking has led to a situation where every seventh death in men and every 13^th^ death in women before age 65 in the EU is caused by alcohol [25].

Clearly, this level of risk is not acceptable by usual standards. This finding of an unacceptable risk from the daily drinking of 20 g can be confirmed by the traditional risk assessment methodology in chemical toxicology, which determines an acceptable daily intake (ADI) based on a threshold on the dose-response curve combined with a safety factor (see WHO International Programme on Chemical Safety for methodology [26]). The ADI for alcohol was determined to be 2.6 g/day based on dose-response modelling of epidemiological data for liver cirrhosis morbidity and mortality [27], which can be interpreted as a “virtually safe dose”. Exceeding this dose, which would be caused by drinking even one standard drink per day, would normally be interpreted as a concern in terms of health, making the food unacceptable for consumption (for example, in cases of additives or pesticides exceeding ADI). Clearly, the ADI also needs to be interpreted in light of the difference between voluntary and involuntary risks. Otherwise, any alcoholic beverage would not be marketable *per se*.

It is harder to estimate the involuntary mortality risk from alcohol exposure, and only a few studies have tried [28,29]. In Australia, there was a yearly burden in 2008 of 367 deaths and almost 14,000 hospitalizations due to drinking by others [30], indicating yearly risks of higher than one in 100,000 deaths, and about 0.5 per 1,000 hospitalizations, the former clearly much higher than the usually accepted involuntary risk of one in one million [3,22]. Thus, it seems that alcohol-attributable voluntary and involuntary mortality risks exceed usual thresholds.

What are the factors leading to this situation? First, alcohol is not internationally regulated as an addictive substance along with illegal drugs, tobacco and pharmaceuticals [31], despite ranking as more harmful to the individual and to society than most of the substances being regulated this way, no matter what criteria are applied [32]. The lack of international regulation started as a historical accident, reflecting a long era of reaction against the alcohol prohibitions imposed in the early 20th century in more than a dozen countries [33]. That alcohol is not internationally regulated also reflects its cultural acceptance among elites in most western societies, and the strong political influence of global alcohol producers [34]. The most recent WHO Expert Committee on Drug Dependence discussed whether alcohol should be considered for coverage by the international drug treaties, and recommended that a pre-review of this should be considered [35]. But it is a long way from this to any effective public health regulation internationally, if only to protect national alcohol policies from further erosion under the international trade agreements.

Second, alcohol is not treated like other food products. While food products need to declare all their ingredients in the EU and North America, alcohol does not. In the US, alcohol was not considered a food in the temperance era, and alcoholic beverages are still regulated by a different government agency (Alcohol and Tobacco Tax and Trade Bureau) from foods (FDA). In Europe, alcoholic beverages are regulated under general food laws (Regulation (EC) No. 178/2002). However, alcoholic beverages have been exempted from the mandatory labelling of the list of ingredients and the nutrition declaration according to Regulation (EU) No 1169/2011 on the provision of food information. Whether alcoholic beverages should in future be covered by the regulation is currently under discussion. Moreover, alcohol is a well-known carcinogen with a causal role in oral cavity, pharynx, larynx, oesophagus, colon, rectum, liver (hepatocellular carcinoma) and female breast cancer [36], and its concentrations in usual daily use in Europe substantially exceed thresholds typically accepted for carcinogens in foods [37]. Interestingly, under California’s Health and Safety Code, a warning sign must be posted in stores selling alcohol, stating that alcohol increases cancer risk [38]. This regulation was established by the Office of Environmental Health Hazard Assessment.

Third, alcohol is often treated ambivalently by the public health community, partly as a generational reaction against temperance movements, which had had strong public health support [39], and also because of the often-overestimated beneficial effect of light to moderate drinking on ischaemic diseases and diabetes (see [18] for an overview). However, these protective effects have already been considered by all of the calculations on alcohol-attributable burden, and the burden reported in the introduction here [6,9] is net burden.

Fourth, the risks of alcohol may not be fully understood by the general public. Surveys in many high-income countries showed that the impact of alcohol on cancer was known only by a minority: 33% in a large general population survey in Canada 2008, [40]; 30% on average in CAMH Monitor - representative surveys for the province of Ontario - in 1996, 2004, 2007, 2012 (personal communication from Ms. A. Ialomiteanu); 38% in the US [41]; 14% in the UK [42]; and only 36% of the EU population “agree totally” that alcohol can increase the risk of cancer [43]. Thus, the acceptable risk threshold by individuals and in societies may be in part based on incomplete information (see [44,45] for risk perception in general; see [46] for a systematic review, how being conscientious of the risks may impact individual decisions and mortality). However, there may be other individual-level variables that impact on risk perception for alcohol, such as personal risks from alcohol being seen as much lower than risks of the same amount of consumption in others [47].

In assessing mortality risk and health burden due to alcohol consumption, one should not overlook that the burden of alcohol goes well beyond the health field and includes social consequences to those around the drinker and to wider society, such as crime, lost productivity, family problems, child neglect or abuse, and social marginalization [48]. An Australian study found that the reported tangible costs from out-of-pocket expenses and time lost because of others’ drinking were of much the same magnitude as the costs to health, social and legal systems of dealing with problems from drinking [30]. While it may prove hard to integrate the metrics of the burden of these social consequences with the health burden, they underline the necessity to change our negligent attitude towards alcohol consumption and its risk. After all, there are policy measures to reduce the risk and burden associated with alcohol [49], which have even been shown to be cost-effective [50].

Even after all of the discussion above, questions remain about why societies appear to accept a higher risk for alcohol than for other voluntary risks. If better information is crucial (see above), the lack of knowledge could be overcome with awareness campaigns and proper content labelling of alcohol, including warnings of health risks, as currently discussed in the EU and in Canada. However, empirical studies showed only limited outcomes, at least in the short run and with respect to drinking behaviour [51]. Can it be that the addictive properties of alcohol cloud the consumers’ ability to assess information and make a free choice [52]? Or may the actual or perceived pleasurable effects of alcohol consumption (that is, benefits) be so high that the informed choice of a mortality risk in the 1:100 range is seen as “reasonable risk” [53], so that there is no pressure from the public for government action, and governments are dissuaded from effective alcohol policies such as raising taxes [54]?

## Summary

Alcohol consumption incurs voluntary and involuntary risks that exceed the risks modern societies in high-income countries are willing to accept for other risk behaviours and factors. This acceptance is exemplified by how alcohol is treated within the food legislation, within the international treaties for psychoactive substances and within public health frameworks. While lack of information about alcohol-attributable risks may play a role, the reasons why alcohol is treated so exceptionally are currently not fully understood.

## Additional file

Additional file 1:
**Risk of dying due to alcohol for different levels of average drinking in six European countries.** Methodology for the calculations.
