# Supplementary material for: Why does society accept a higher risk for alcohol than for other voluntary or involuntary risks?
Source: BMC Med. 2014 Oct 21;12:189. doi: 10.1186/s12916-014-0189-z (PMC4203927; doi:10.1186/s12916-014-0189-z)
Supplement: Additional file 1: — Risk of dying due to alcohol for different levels of average drinking in six European countries. Methodology for the calculations. [file 12916_2014_189_MOESM1_ESM.docx]

# Additional file 1

# Risk of dying due to alcohol for different levels of average drinking in six European countries. Methodology for the calculations.

## Causes of death causally impacted by different levels of alcohol consumption

Table S1 gives an overview of mortality risks causally impacted by alcohol consumption. The list here is identical to the causes of deaths, used as a basis of the Global Status Report on Alcohol and Health [[1](#_ENREF_1)], and to the causes used in the Global Burden of Disease Study [[2](#_ENREF_2)] except the definition of alcohol use disorders and the impact on HIV/AIDS medication [[3](#_ENREF_3)]. The latter is listed but was not used in our calculations as these risks tend to be very low due to relative small numbers. In any case, the risk would increase if this is added

### Table S1: Categories of alcohol-attributable disease and the sources used for determining risk relations

| **Condition** | | **ICD 10 Code** | **Sources on risk relations (for AAF)** |
| --- | --- | --- | --- |
| **Infectious and parasitic diseases** | |  |  |
|  | Tuberculosis | A15-A19 | [[4](#_ENREF_4)]; for causal relationship see: [[5](#_ENREF_5)] |
|  |  |  |  |
| **Human immunodeficiency virus/ Acquired immune deficiency syndrome** | | B20-B24 | [[3](#_ENREF_3)] for estimate on the impact of alcohol on worsening the disease course via disrupting the medication schedule –> not relevant for Europe because of small numbers as cause of deaths |
|  |  |  |  |
| **Malignant neoplasms** | |  |  |
|  | Mouth and oropharynx cancers | C00-C14 | [[6](#_ENREF_6), [7](#_ENREF_7)](based on Relative Risks from [[8](#_ENREF_8)]) |
|  | Esophageal cancer | C15 | [[6](#_ENREF_6), [7](#_ENREF_7)] (based on Relative Risks from [[8](#_ENREF_8)]) |
|  | Liver cancer | C22 | [[6](#_ENREF_6), [7](#_ENREF_7)] (based on Relative Risks from [[8](#_ENREF_8)]) |
|  | Laryngeal cancer | C32 | [[6](#_ENREF_6), [7](#_ENREF_7)] (based on Relative Risks from [[8](#_ENREF_8)]) |
|  | Breast cancer | C50 | [[6](#_ENREF_6), [7](#_ENREF_7)] (based on Relative Risks from [[8](#_ENREF_8)]) |
|  | Colon cancer | C18 | [[6](#_ENREF_6), [7](#_ENREF_7)] (Combined risk taken from [[9](#_ENREF_9)] |
|  | Rectal cancer | C20 |  |
| **Diabetes** | |  |  |
|  | Diabetes mellitus | E10-E14 | [[10](#_ENREF_10)] |
| **Neuro-psychiatric conditions** | |  |  |
|  | Alcoholic psychoses (part of alcohol use disorders = AUD) | F10.0, F10.3-F10.9 | Special analysis, see text |
|  | Alcohol abuse (part of AUD) | F10.1 |  |
|  | Alcohol dependence (part of AUD) | F10.2 |  |
|  | Epilepsy | G40-G41 | [[11](#_ENREF_11)] |
| **Cardiovascular disease** | |  |  |
|  | Hypertensive disease | I10-I15 | [[12](#_ENREF_12)] |
|  | Ischemic heart disease | I20-I25 | [[13](#_ENREF_13)]; pattern was only included into the calculations for RR < 1 in categories of 60g and above, which were set to 1 (based on [[14](#_ENREF_14)]) |
|  | Cardiac arrhythmias | I47-I49 | [[15](#_ENREF_15)] |
|  | Ischemic stroke | I60-I62 | [[16](#_ENREF_16)]; consistent with ischemic heart disease, pattern was only included into the calculations for RR<1 in categories of 60g and above, which were set to one |
|  | Hemorrhagic and other non-ischemic stroke | I63-I66 | [[16](#_ENREF_16)] |
| **Digestive diseases** | |  |  |
|  | Cirrhosis of the liver | K70, K74 | [[17](#_ENREF_17)] |
|  | Acute and chronic pancreatitis | K85, K86.1 | [[18](#_ENREF_18)] |
| **Respiratory infections** | |  |  |
|  | Lower respiratory infections | J10–J18, J20–J22 | [[19](#_ENREF_19)] |
| **Conditions arising during the prenatal period** | |  |  |
|  | Low birth weight: as defined by the GBD | P05-P07 | [[20](#_ENREF_20)] -> cause of death not included, as we only included the harm of drinking to drinkers. |
| **Unintentional injuries** | |  |  |
|  | Motor vehicle accidents | § | [[8](#_ENREF_8)] |
|  | Poisonings | X40-X49 | [[8](#_ENREF_8)] |
|  | Falls | W00-W19 | [[8](#_ENREF_8)] |
|  | Fires | X00-X09 | [[8](#_ENREF_8)] |
|  | Drowning | W65-W74 | [[8](#_ENREF_8)] |
|  | Other Unintentional injuries | †Rest of V-series and W20-W64, W 75-W99, X10-X39, X50-X59, Y40-Y86, Y88, and Y89 | [[8](#_ENREF_8)] |
| **Intentional injuries** | |  | [[8](#_ENREF_8)] |
|  | Self-inflicted injuries | X60-X84 and Y87.0 | [[8](#_ENREF_8)] |
|  | Homicide | X85-Y09, Y87.1 | [[8](#_ENREF_8)] |
|  | Other intentional injuries |  | [[8](#_ENREF_8)] |
| § V021–V029, V031–V039, V041–V049, V092, V093, V123–V129, V133–V139, V143–V149, V194–V196, V203–V209, V213–V219, V223–V229, V233–V239, V243–V249,V253–V259, V263–V269, V273– V279, V283–V289, V294–V299, V304–V309, V314–V319, V324–V329, V334–V339, V344–V349, V354–V359, V364–V369, V374–V379, V384–V389, V394–V399, V404–V409, V414–V419, V424–V429, V434–V439, V444–V449, V454–V459, V464– V469, V474–V479, V484–V489, V494–V499, V504–V509, V514–V519, V524–V529, V534–V539, V544–V549, V554–V559, V564–V569, V574–V579, V584–V589, V594–V599, V604–V609, V614–V619, V624–V629, V634–V639, V644–V649, V654– V659, V664–V669, V674–V679, V684–V689, V694–V699, V704–V709, V714–V719, V724–V729, V734–V739, V744–V749, V754–V759, V764–V769, V774–V779, V784–V789, V794–V799, V803–V805, V811, V821, V830–V833, V840–V843, V850– V853, V860–V863, V870–V878, V892. †Rest of V = V-series MINUS §. | | | |

## Selection of operationalizations and methodology

Average level of daily alcohol consumption was taken in steps of 10g of pure alcohol up to 100g. For all alcohol-attributable disease categories we had meta-analyses on dose-response relationships as listed in Table S1. The relative risks were determined by evaluating the risk functions at 10g, 20g, 30g, … up to 100g. The corresponding alcohol-attributable fractions were calculating by the following formula [[21](#_ENREF_21)]:

$$AAF=\frac{RR\left( x \right)-1}{RR(x)}$$

Where RR(x) is the relative risk for an average daily consumption of x g/day for a given disease. This formula derives directly from its general form [[22](#_ENREF_22)].

$$AAF= \frac{P_{abs}+P_{form}{RR}_{form}+\int_{0}^{\infty} P\left( x \right)RR\left( x \right)dx- 1}{P_{abs}+P_{form}{RR}_{form}+\int_{0}^{\infty} P\left( x \right)RR\left( x \right)dx}$$

when the entire population is assumed to be drinking an average of x g/day.

The absolute risk of dying from an alcohol-attributable death for 2012 for any given sex-age group could be expressed as follows:

$${Risk.Death.AA}_{zq} =\frac{\sum_{i=1}^{n} {(AAF}_{izq}*{Deaths}_{iz})+{Deaths}_{AUD}*\frac{{Prev.AD}_{Couterfactualq}}{{Prev.AD}_{Current}}}{{Population}_{z}}$$

where the risk of dying from an alcohol-attributable death assumes an alcohol consumption level of q for a person in age group z divided by the population for age group z. The number of alcohol-attributable deaths is the sum of all deaths of cause of death categories causally related to alcohol i (where n equals the total number of cause of death categories causally related to alcohol; see Table S1) for age group z multiplied by the alcohol-attributable fraction for cause of death I, age group z and alcohol consumption level q added to the number of deaths caused by Alcohol Use Disorders (AUD) multiplied by the product of the prevalence of alcohol dependence (AD) under a counterfactual scenario where everyone consumes q grams of alcohol divided by the prevalence of AD under current conditions.

Data on the prevalence of AD for countries under current conditions were obtained from [[23](#_ENREF_23)] and data for counterfactual scenarios were obtained from alcohol consumption data among people with AD from the recent APC-Study (not yet published). Age groups were split into five-year intervals starting at 15 years of age. Population data and mortality data were obtained from the World Health Organization [[[1](#_ENREF_1)] based on Global Health Estimates: <http://www.who.int/healthinfo/global_burden_disease/en/> )

All calculations were performed by age group separately for all countries of interest and for men and women in each country. the risks for diseases other than AUD are based on alcohol-attributable fractions, i.e., on the proportion which is caused by alcohol and which would disappear under the assumption of no alcohol consumption (for explaining the methodology and background see [[21](#_ENREF_21), [24](#_ENREF_24), [25](#_ENREF_25)]; for alcohol see [[22](#_ENREF_22)]). This model could not be employed for AUD, as these are 100% alcohol attributable by definition. So a different methodology had to be applied, essentially multiplying the probability of alcohol dependence, given different levels of drinking (see above). However, there are additional problems with the mortality from AUD: while AUDs are associated with a high level of mortality ([[26-28](#_ENREF_26)], they usually do not appear as a major underlying cause of death in European or global statistics for many reasons including stigmatization (i.e., insurance in some European countries will not pay, if death is “self-afflicted” by alcoholism; about the stigmatization associated with AUD see [[29](#_ENREF_29)]).

All analyses were conducted first subtracting the current impact of alcohol consumption and then re-adding the alcohol-attributable part based on the new drinking patterns by applying the attributable fractions (see above). The sex-, age- and disease-specific alcohol-attributable part of total mortality was taken from the Global Status Report [[1](#_ENREF_1)]. While this procedure removes some of the impact by patterns of drinking (e.g., some of the impact on injury – [[30](#_ENREF_30)]– and ischemic heart disease – [[31](#_ENREF_31)]), we suspect that other impacts of patterns of drinking can be found in the country-specific contribution of causes of death remaining after the subtraction of alcohol. An example here would be the high proportion of misclassified alcohol poisoning deaths in Estonia [[32](#_ENREF_32), [33](#_ENREF_33)]. In other words: the country differences in risk allow us to check on the stability of results from different perspectives.

Lastly an analysis was performed where the risk of an alcohol-attributable death for a given year was adjusted based on the risk of dying from a non-alcohol-attributable death prior to that year. This adjustment was performed using the following formula:

$${POP}_{i}=\sum_{i=0}^{n} {POP}_{i-1}-({RiskDeathsNonAA}_{i}+{RiskDeathsAA}_{i}*{POP}_{i-1} )$$

where POP_i_ is the population left at the end of age i (i starts at 1 for 0 years into a persons life course and decreases as age increases) RiskDeathsNonAA_I_ represents the risk of a death from a non-alcohol-attributable cause for a person age i, and RiskDeathsAA_i_ represents the risk of a death from an alcohol-attributable cause for a person age i. Using this formula we can then calculate the total risk of an alcohol attributable death as follows:

$${CumulativeRiskAlcohol}_{i}= \sum_{i=0}^{n} {{CumulativeRiskAlcohol}_{i-1}+(RiskDeathsNonAA}_{i}*{POP}_{i-1} )$$

where CumulativeRiskAlcohol represents the cumulative lifetime risk of dying from an alcohol-attributable death at age i in a person’s life course.

Reference List

1. World Health Organization: **Global status report on alcohol and health**, Geneva, Switzerland: World Health Organization; 2014.

2. Lim SS, Vos T, Flaxman AD, Danaei G, Shibuya K, Adair-Rohani H, Amann M, Anderson HR, Andrews KG, Aryee M *et al*: **A comparative risk assessment of burden of disease and injury attributable to 67 risk factors and risk factor clusters in 21 regions, 1990–2010: a systematic analysis for the Global Burden of Disease Study 2010** *Lancet* 2012, **380**:2224-2260.

3. Gmel G, Shield K, Rehm J: **Developing a methodology to derive alcohol-attributable fractions for HIV/AIDS mortality based on alcohol's impact on adherence to antiretroviral medication**. *Popul Health Metr* 2011, **9**(1):5.

4. Lönnroth K, Williams B, Stadlin S, Jaramillo E, Dye C: **Alcohol use as a risk factor for tuberculosis - a systematic review**. *BMC Public Health* 2008, **8**:289.

5. Rehm J, Samokhvalov AV, Neuman MG, Room R, Parry CD, Lönnroth K, Patra J, Poznyak V, Popova S: **The association between alcohol use, alcohol use disorders and tuberculosis (TB). A systematic review**. *BMC Public Health* 2009, **9**(1):450.

6. Baan R, Straif K, Grosse Y, Secretan B, El Ghissassi F, Bouvard V, Alteri A, Cogliano V, On behalf of the W. H. O. International Agency for Research on Cancer monograph working group: **Carcinogenicity of alcoholic beverages**. *Lancet Oncology* 2007, **8**(4):292-293.

7. International Agency for Research on Cancer: **IARC Monograph 96 on the Evaluation of Carcinogenic Risks to Humans. Alcoholic beverage consumption and ethyl carbamate (urethane).** Lyon, France: International Agency for Research on Cancer (IARC); 2010.

8. Corrao G, Bagnardi V, Zambon A, La Vecchia C: **A meta-analysis of alcohol consumption and the risk of 15 diseases**. *Prev Med* 2004, **38**:613-619.

9. Bagnardi V, Rota M, Botteri E, Tramacere I, Islami F, Scotti L, Turati F, Galeone C, Bellocco R, Boffetta P: **Alcohol consumption and site-specific cancer risk: a comprehensive dose-response meta-analysis**. *Ann Oncol* in press.

10. Baliunas D, Taylor B, Irving H, Roerecke M, Patra J, Mohapatra S, Rehm J: **Alcohol as a risk factor for type 2 diabetes - A systematic review and meta-analysis**. *Diabetes Care* 2009, **32**(11):2123-2132.

11. Samokhvalov AV, Irving H, Mohapatra S, Rehm J: **Alcohol consumption, unprovoked seizures and epilepsy: a systematic review and meta-analysis**. *Epilepsia* 2010, **51**(7):1177-1184.

12. Taylor B, Irving HM, Baliunas D, Roerecke M, Patra J, Mohapatra S, Rehm J: **Alcohol and hypertension: gender differences in dose-response relationships determined through systematic review and meta-analysis**. *Addiction* 2009, **104**(12):1981-1990.

13. Roerecke M, Rehm J: **The cardioprotective association of average alcohol consumption and ischaemic heart disease: a systematic review and meta-analysis**. *Addiction* 2012, **107**(7):1246-1260.

14. Roerecke M, Rehm J: **Irregular heavy drinking occasions and risk of ischemic heart disease: a systematic review and meta-analysis**. *Am J Epidemiol* 2010, **171**(6):633-644.

15. Samokhvalov AV, Irving HM, Rehm J: **Alcohol as a risk factor for atrial fibrillation: a systematic review and meta-analysis**. *Eur J Cardiovasc Prev Rehabil* 2010, **17**(6):706-712.

16. Patra J, Taylor B, Irving H, Roerecke M, Baliunas D, Mohapatra S, Rehm J: **Alcohol consumption and the risk of morbidity and mortality from different stroke types - a systematic review and meta-analysis**. *BMC Public Health* 2010, **10**(1):258.

17. Rehm J, Taylor B, Mohapatra S, Irving H, Baliunas D, Patra J, Roerecke M: **Alcohol as a risk factor for liver cirrhosis - a systematic review and meta-analysis**. *Drug Alcohol Rev* 2010, **29**(4):437-445.

18. Irving HM, Samokhvalov A, Rehm J: **Alcohol as a risk factor for pancreatitis. A systematic review and meta-analysis**. *JOP* 2009, **10**(4):387-392.

19. Samokhvalov AV, Irving HM, Rehm J: **Alcohol consumption as a risk factor for pneumonia: systematic review and meta-analysis**. *Epidemiol Infect* 2010, **138**(12):1789-1795.

20. Patra J, Bakker R, Irving H, Jaddoe VWV, Malini S, Rehm J: **Dose-response relationship between alcohol consumption before and during pregnancy and the risks of low birthweight, preterm birth and small for gestational age (SGA)-a systematic review and meta-analyses**. *BJOG: International Journal of Obstetrics and Gynaecology* 2011, **118**(12):1411-1421.

21. Rockhill B, Newman B: **Use and misuse of population attributable fractions**. *Am J Public Health* 1998, **88**:15-19.

22. Rehm J, Kehoe T, Gmel G, Stinson F, Grant B, Gmel G: **Statistical modeling of volume of alcohol exposure for epidemiological studies of population health: the example of the US**. *Popul Health Metr* 2010, **8**:3.

23. Rehm J, Shield KD, Rehm MX, Gmel G, Jr., Frick U: **Alcohol consumption, alcohol dependence, and attributable burden of disease in Europe: potential gains from effective interventions for alcohol dependence,** Toronto, Canada: Centre for Addiction and Mental Health; 2012.

24. Walter SD: **The estimation and interpretation of attributable risk in health research**. *Biometrics* 1976, **32**:829-849.

25. Ezzati M, Lopez A, Rodgers A, Murray CJL: **Comparative quantification of health risks. Global and regional burden of disease attributable to selected major risk factors**, Geneva, Switzerland: World Health Organization; 2004.

26. Roerecke M, Rehm J: **Alcohol use disorders and mortality - A systematic review and meta-analysis**. *Addiction* 2013, **108**(9):1562-1578.

27. Roerecke M, Rehm J: **Cause-specific mortality risk in alcohol use disorder treatment patients: a systematic review and meta-analysis**. *Int J Epidemiol* 2014, **43**(3):906-919.

28. Rehm J, Dawson D, Frick U, Gmel G, Roerecke M, Shield KD, Grant B: **Burden of disease associated with alcohol use disorders in the United States** *Alcohol Clin Exp Res* 2014, **38**(4):1068-1077.

29. Schomerus G, Lucht M, Holzinger A, Matschinger H, Carta MG, Angermeyer MC: **The stigma of alcohol dependence compared with other mental disorders: a review of population studies**. *Alcohol Alcohol* 2011, **46**(2):105-112.

30. Shield KD, Gmel G, Jr., Patra J, Rehm J: **Global burden of injuries attributable to alcohol consumption in 2004: a novel way of calculating the burden of injuries attributable to alcohol consumption**. *Popul Health Metr* 2012, **10**:9.

31. Shield KD, Parry C, Rehm J: **Chronic diseases and conditions related to alcohol use** *Alcohol Res* 2013, **35**(2):155-171.

32. Ringmets I, Tuusov J, Lang K, Väli M, Pärna K, Tõnisson M, Helander A, McKee M, Leon DA: **Alcohol and premature death in Estonian men: a study of forensic autopsies using novel biomarkers and proxy informants**. *BMC Public Health* 2012, **12**:146.

33. Rahu K, Palo E, Rahu M: **Diminishing trend in alcohol poisoning mortality in Estonia: reality or coding peculiarity?** *Alcohol Alcohol* 2011, **46**(4):485-489.
